# Comparison of tissue response and lifting effect induced by non-absorbable elastic thread and commercialized threads in rat model

**DOI:** 10.1093/rb/rbae069

**Published:** 2024-06-17

**Authors:** Dae Hyung Lee, Yeji Choi, Mi Hee Lee, Jong-Chul Park

**Affiliations:** Advanced Medical Device R&D Center, HansBiomed Co., Ltd, Seoul 05836, Republic of Korea; Cellbiocontrol Laboratory, Department of Medical Engineering, Yonsei University College of Medicine, Seoul 03722, Republic of Korea; Advanced Medical Device R&D Center, HansBiomed Co., Ltd, Seoul 05836, Republic of Korea; Cellbiocontrol Laboratory, Department of Medical Engineering, Yonsei University College of Medicine, Seoul 03722, Republic of Korea; Cellbiocontrol Laboratory, Department of Medical Engineering, Yonsei University College of Medicine, Seoul 03722, Republic of Korea

**Keywords:** elasticity, lifting, rejuvenation, suture thread

## Abstract

As we age, our skin loses elasticity and wrinkles form. To prevent this, most people try to improve skin wrinkles by performing procedures such as fillers, and absorbable lifting threads. Another way to solve this structural problem is to use an elastic thread. Although elastic sutures made of polymer materials (such as silicone) are widely used, data regarding their properties and potential effectiveness are lacking. This study aimed to investigate the effects of inserting non-absorbable elastic threads, with different manufacturing requirements and methods, on the skin and subcutaneous tissue. In this study, non-absorbable elastic threads ELATENS and Elasticum using different manufacturing methods were compared. Each thread was transplanted into the subcutaneous layer of the back of a rat to induce wrinkles. After inducing wrinkles in the skin of rat, the degree of skin maintenance by each thread and the thickness of the capsule formed around the suture were measured. Through *ex-vivo* experiments on each thread, the fixation force in the tissue was confirmed. In a comparison of inflammatory response and collagen formation through histological analysis, it was confirmed that there was no significant difference from the equivalent comparative product. In conclusion, the degree of encapsulation between tissues and collagen fiber formation for improving skin wrinkles was superior in elastic threads compared to non-elastic threads. It is believed that this provides certain elasticity to the skin layer and can induce cell influx to improve wrinkles.

## Graphical Abstract

**Figure:**
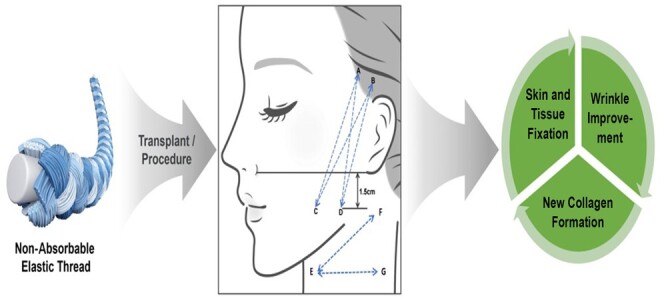

## Introduction

Owing to an increase in the percentage of the elderly population, anti-aging procedures are garnering a lot of attention. Aging is a biological phenomenon induced by internal and external factors, and the most prominent characteristics of aging appear on the facial skin. Among the available anti-aging techniques, invasive surgical procedures have been performed frequently, they require an extensive incision in the musculoaponeurotic system and a long recovery period. Moreover, non-invasive or minimally invasive surgical procedures such as laser treatment, ultrasound therapy, dermal filler injections and micro fat grafting are being performed, but these have several limitations in terms of overcoming skin problems such as wrinkles that have been already formed and sagging skin in the lower part of the face [1].

Among skin anti-aging procedures, a superficial musculoaponeurotic system (SMAS) facelift has been considered the gold standard for facial structure correction. However, this procedure is very invasive and involves extensive dissection, including detaching the skin from SMAS; it also has disadvantages such as a long recovery period and postoperative complications [2, 3]. In addition, as the skin of Asians is thicker than the skin of White people, this procedure is not effective for lifting thick and aging skin and soft tissues of Asians [4, 5].

Facial contouring surgery to remove facial wrinkles using a special thread, i.e. a facelift, which is one of the minimally invasive surgical procedures, was developed as a new technique to compensate for the limitations of the existing facelift procedures [6, 7]. Thread lifting is a minimally invasive procedure to lift and rearrange the facial skin and the loose soft tissue below; it has advantages such as invisible scars, short recovery period and fewer side effects [8, 9].

An elastic suture thread was used as a special thread in this study. The elastic suture technique has already been reported by Raskin in 1993, and it arose from the notion that an elastic band fixed to the skin at the edges of a wound produces tension so as to provide skin elasticity. The anatomical and histological structure of the skin gives it viscoelasticity; therefore, if consistent traction is applied to the skin, skin extension may gradually occur. This is called the ‘creep phenomenon’. As a method to solve this problem, an elastic suture can prevent ischemic necrosis at the edges of the wound by evenly distributing elastic traction to the entire tissues [10].

An example of such elastic suture is the use of Spring Thread^®^ (1st SurgiConcept 96 Rue de Pont, Rompu 59200 Tourcoing, France), which is a suture thread with elasticity and flexibility provided by a silicone thread as a biocompatible composite material. It has elasticity similar to that of the skin. Pain and bruising decreased when Spring Thread^®^ was used owing to the compensatory effect against creep induced by the previous suture threads. It has been reported that these effects are caused by stable and consistent skin traction resulting from the thread’s elasticity and flexibility [11, 12].

Recently, Elasticum^®^ (Korpo SRL, Genova, Italy), a suture thread with similar elasticity, was used in thread-lifting procedures. Unlike other threads, Elasticum^®^ has been reported to stretch along as facial muscles move owing to its elasticity, which contributes to natural-looking facelift results [13–15].

Based on this, a new form of a non-absorbable elastic thread (ELATENS; HansBiomed Co. Ltd, Seoul, Korea) was developed. ELATENS is a non-absorbable, elastic suture thread and is comprised of an elastomer in the core and a surrounding enveloping layer of non-elastic threads. In our previous comparative evaluation study, this special thread and Elasticum had been compared and evaluated in terms of their biomechanical properties and biocompatibility [16–18].

This study aimed to investigate the effects of inserting non-absorbable elastic threads, on the skin and subcutaneous tissue. The detailed objectives were as follows:

1. To determine the force required to pull out a thread implanted into the dorsal subcutaneous muscle layer of rats.
2. To determine the extent to which the pulled skin is maintained over time by a thread implanted into the dorsal subcutaneous muscle layer of rats.
3. To conduct histological analysis of the rats’ skin into which a thread was implanted, and then determine the degree of capsule formed around the thread [19].
4. Determine the amount of friction required to pull out a thread implanted into the leg muscles of rats.
5. To conduct histological analysis of the rats’ skin into which a thread was implanted, and then determine the extent of inflammatory cell invasion and collagen formation around the thread.

## Materials and methods

### Preparation of test materials

ELATENS, non-absorbable elastic threads were fabricated by braiding PET, a non-elastic thread, on a silicone elastomer thread. Two types of commercially available threads were used to fabricate ELATENS for this study—an elastic thread with silicone (silicone elastomer; MED-4755, Nusil, USA) as an elastomer, and a non-elastic thread made of polyester (PET; 150 denier/48-filament high tenacity polyester yarn, textile development associates, USA). Briefly introduce the manufacturing method, it is manufactured by placing an elastic silicone thread in the center and braiding PET, an inelastic thread, into an even number of strands on the outside to form a layer. For the current experiment, the Elasticum^®^, a non-absorbable elastic suture material that is made of silicone and sheathed with polyester, served as a control material [14, 15]. It is distinguished by differences in the surface, which is formed depending on the ways in which PET is braided. The detailed specimen fabrication requirements are shown in Table 1 [16]. In the case of these elastic threads, the outer covering is made of non-elastic threads (PET). In the Supplementary Figure, SEM images of the surface and cross-section of ELASTAN and Elasticum were shown. Therefore, for experimental comparison, non-elastic threads (PET), the outer covering excluding the silicone threads in the center, were used as a control material.

**Table 1.** Conditions and standardization of non-absorbable elastic threads

	Silicone		PET		Diameter (mm)
	Diameter (mm)	pcs	Diameter (mm)	pcs	
ELATENS	0.5	2	0.05	8	1.20
Elasticum	0.5	2	0.20	2	1.20
PET only	—	—	0.05	8	0.50

PET, polyester.

### Subcutaneous transplant of test materials

Twelve-week-old Sprague Dawley rats were used for the *in vivo* experiment. For animal testing, an animal laboratory of an institution approved by the Korea Food and Drug Administration (KFDA) (Hans Biomed Animal Testing Facility, approval number: 648) was used. The animals weighing 250–300 g were kept under the 12/12 h light/dark cycle and maintained in pathogen-free conditions with free access to food and drinking water. All the procedures for animal testing were conducted in accordance with the KFDA guidelines for the Care and Use of Laboratory Animals.

**Figure 1.**
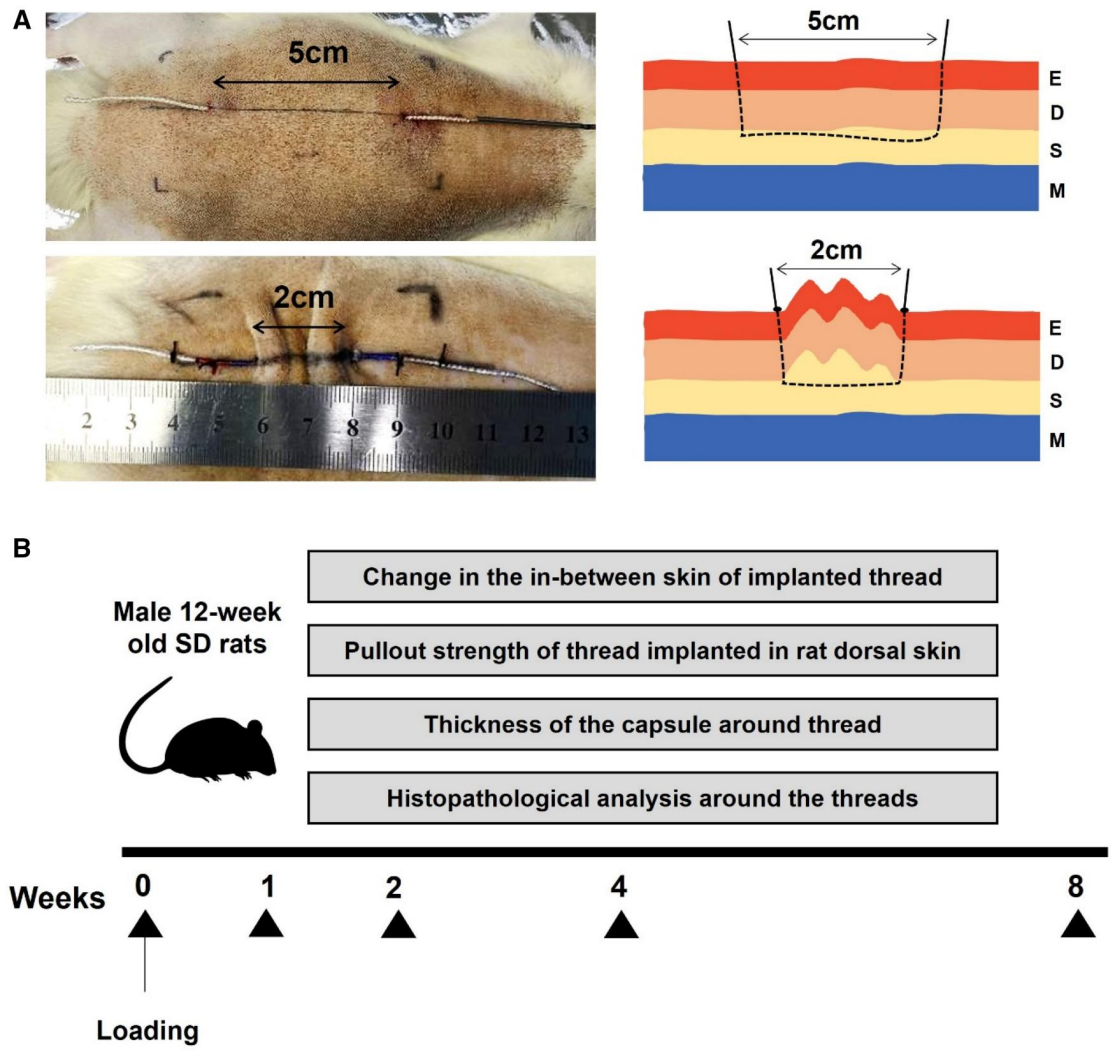
Overall experimental design of the study. (**A**) Description of how to wrinkle and fix the dorsal skin and subcutaneous tissue of a rat. (**B**) Analysis schedule at weeks 1, 2, 4 and 8 after transplantation of each thread. E, epidermis; D, dermis; S, subcutaneous tissue; M, muscle.

The experiments were performed on 54 Sprague Dawley rats under inhalation anesthesia. Hair on the dorsal skin of the rats was removed, and the area was disinfected with alcohol. Subsequently, a 4 cm × 8 cm rectangle was drawn on the skin. A suture needle attached to the thread was inserted into the skin and through the subcutaneous tissue on the vertical median line of the drawn rectangle. Depending on the use of different threads, the rats were divided into three groups—ELATENS (HansBiomed Co. Ltd, Seoul, Korea), Elasticum (Korpo SRL, Genova, Italy) and polyester (PET) yarn (Textile Development Associates Inc., Brookfield, CT, USA). Each thread corresponding to the group was implanted into the dorsal subcutaneous layer of the rat. The skin and subcutaneous tissue between the insertion point and exit point of the thread were wrinkled from 50 to 25 mm and fixed to prevent the skin tissue from moving. The remaining suture thread outside the skin tissue was fixed using a 2-0 silk suture (Ethicon) to keep it from pulling out. After the implantation of each thread, analyses were performed at weeks 1, 2, 4 and 8 (Figure 1).

### Change in the in-between skin of the implanted thread

After insertion, the thread was pulled from both ends to make the entire skin creased. Then, we determined the extent to which the length of the pulled skin was maintained over time. The length of the skin between the insertion and exit points of the threads in three groups was measured using vernier calipers immediately after insertion and at weeks 1, 2 and 4.

### Pullout strength of thread implanted in rat dorsal skin

To determine the reason behind the skin and subcutaneous tissue being pulled by a thread, we measured the amount of force required to pull out the thread implanted into the tissues. To prevent the thread from being stuck in the hair of rats while being pulled out, the hair around the area where the thread was hanging was shaved after the rats were anesthetized. One end of a 2-0 silk suture (Ethicon), which had been fixed beforehand to the part of the thread outside the skin tissue, was cut off to obtain only one end hanging on the skin tissue. The end of the hanging thread was held using the grip of a tensiometer, and then the thread was slowly pulled so that it could come out of the skin. At that point, the maximum tensile strength was measured using portable tensile testing equipment (IMADA #EPXMRF96).

### Thickness of the capsule around thread implanted in rat dorsal skin

After the threads were inserted into the skin and subcutaneous tissue, the thickness of the capsule formed around the thread was measured at weeks 1, 2 and 4. Tissues, along with threads, were collected and fixed in 10% formalin for 6–12 h. Subsequently, they were embedded in paraffin and cut into 6-mm-thick micro-sectioned pieces for immunohistochemistry (IHC) analysis. First, hematoxylin–eosin (Sigma-Aldrich, USA) staining was performed for contrast staining, and alpha-smooth muscle actin (α smooth muscle actin, Abcam cat# ab5694) was stained to compare capsule thickness. Following mounting with a gel mounting medium, the slides were observed using an optical microscope (Zeiss, Axio observer).

### *Ex-vivo* studies on the frictional behavior of the thread

To determine the amount of friction produced by the thread within the tissues, threads were pierced into the leg muscles of rats, and the force of friction was analyzed using a universal material testing machine (Instron Calibration Laboratory, USA) according to the methods of the American Society for Testing and Materials and D2256 (Standard Test Method for Tensile Properties of Yarns by the Single-Strand Method). Specimens were fixed with 10 mm of the thread remaining up to the grip after 50 mm of the thread fully penetrated the tissues. The tensile test speed rate was set at 50 mm/min for measurement, which was performed at a load of 100 N [20, 21]. Three sets of specimens were measured, and the mean values were used for further analysis.

### Histopathological analysis—immunohistochemistry

Following the insertion of threads into rat tissues, IHC was performed for analyzing inflammation around the threads at weeks 1, 4 and 8. Slides of micro-sectioned tissues were treated with xylene and deparaffinized. Subsequently, they were processed via a hydration process in the order of 100% alcohol, 90% alcohol, 80% alcohol and 70% alcohol, following which they were washed with distilled water. Anti-CD3 (Abcam cat#16669) and anti-CD163 (Abcam cat#182422) antibodies were used as the primary antibodies, and anti-rabbit IgG (Abcam cat# ab6721) and anti-mouse IgG (Abcam cat# ab97023) antibodies were used as the secondary antibodies. Following mounting with a gel mounting medium, the slides were observed using an optical microscope.

### Histopathological analysis—Masson’s trichrome and Herovici’s collagen staining

Following the insertion of threads into rat tissues, Masson’s trichrome and Herovici’s collagen staining were performed to identify collagen formation around the threads at weeks 1, 4 and 8. For Masson’s trichrome staining, deparaffinized tissue slides were treated with a mordant, i.e. Bouin’s solution, for 1 h at 56°C and then washed. Following this, Weigert’s iron hematoxylin solution was used for nuclear staining and Biebrich scarlet-acid fuchsin solution was used for cytoplasmic staining. Subsequently, through fractionation was performed using a phosphomolybdic-phosphotungstic acid solution, and the collagen fibers were stained with aniline blue solution. In addition, Herovici’s staining, using Herovici’s Collagen Stain Kit (American MasterTech #KTHER), was performed to identify the subtypes of collagen. Following mounting with a gel mounting medium, the slides were observed using an optical microscope.

### Statistical analysis

Every assay was conducted with *n* = 3 per group. IBM SPSS Statistics 26 (IBM, Armonk, NY, USA) was used for the statistical analysis, and the results are displayed as mean ± standard deviation. The one-way analysis of variance with Tukey’s *post hoc* and Kruskal–Wallis *H*-test with a pairwise comparison was performed to see if differences were significant. The following symbols were used to represent the data, respectively: (*) for probability less than 0.05 (*P* < 0.05), (**) for *P* < 0.01, (***) for *P* < 0.005 and (****) for *P* < 0.001. The significance of the difference was also determined using a Student’s *t*-test, with *P* < 0.05 signifying that the difference was significant. The following symbols were used to represent the data, respectively: (*) for *P* < 0.05, (**) for *P* < 0.01 and (***) for *P* < 0.001.

## Results

### Rat dorsal skin—gross examination and changes in the in-between skin

As shown in Figure 1, after the suture thread was inserted into the dorsal skin and subcutaneous tissue of rats, the analysis was performed at weeks 1, 2, 4 and 8.

**Figure 2.**
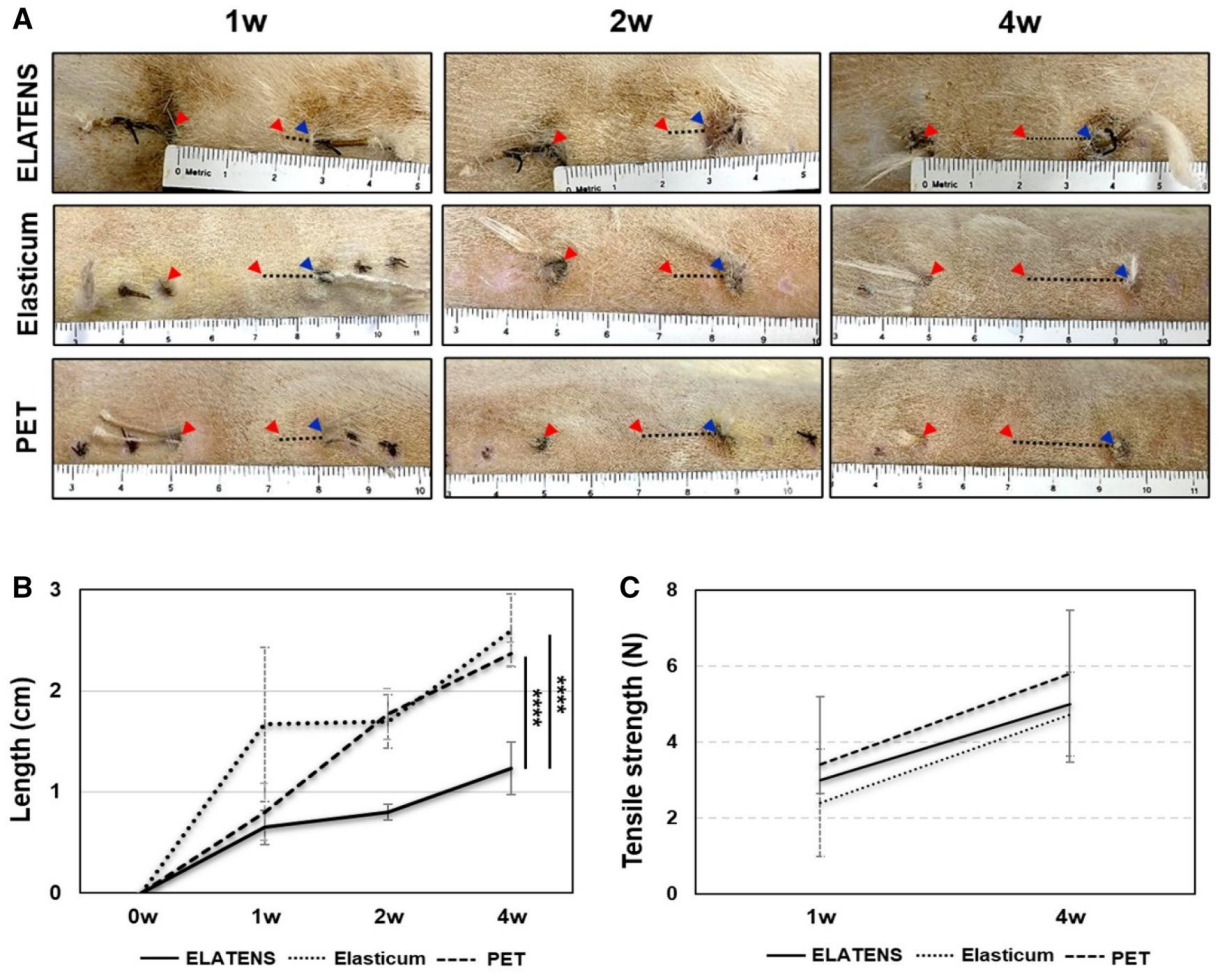
Analysis of tensile strength and length of skin stretched with the thread over time of thread inserted into the rats dorsal skin. (**A**) Representative gross image. The increased length was measured during weeks 1, 2 and 4. Red arrowheads indicate wrinkle length at the beginning of implantation, and blue arrowhead indicate length increase with the post-implantation period. Top: ELATENS, middle: Elasticum, bottom: PET. (**B**) Comparison of the average values of lengths increased during 0, 1, 2 and 4 weeks. Solid: ELATENS, small dotted line: Elasticum, large dotted line: PET. (**C**) The average values of pullout strength at weeks 1 and 4 after transplantation were compared. Solid: ELATENS, small dotted line: Elasticum, large dotted line: PET.

As the skin was creased to shrink from 5 to 2 cm, it could stretch from a minimum of 0 cm to a maximum of 3 cm. Following visual evaluation at each time point (Figure 2A), the mean length of the stretched skin was compared among the different thread types. The total length to which the skin stretched in the first week was as follows: ELATENS: 0.65 ± 0.07 cm, Elasticum: 1.67 ± 0.05 cm and PET: 0.80 ± 0.09 cm. The total length to which the skin stretched in the second week was as follows: ELATENS: 0.80 ± 0.08 cm, Elasticum: 1.70 ± 0.09 cm and PET: 1.77 ± 0.09 cm. The total length to which the skin stretched in the fourth week was as follows: ELATENS: 1.23 ± 0.07 cm, Elasticum: 2.60 ± 0.13 cm and PET: 2.37 ± 0.09 cm. As a result of confirming that the total length was maintained up to 4 weeks, the comparison between groups was statistically significant (*P* < 0.001). Compared with other threads at weeks 1, 2 and 4, ELATENS maintained the length of the pulled skin most effectively (Figure 2A and B).

### Rat dorsal skin—pullout strength

Pullout strength was measured while one end of the thread was cut off and the other end was pulled. The measurement was performed at weeks 1 and 4 after the suture threads were inserted into the dorsal skin and subcutaneous tissue of rats. A large force was needed for pulling PET, ELATENS and Elasticum (in that order): 3.47 ± 0.21 N for PET, 3.03 ± 0.15 N for ELATENS and 2.47 ± 0.21 N for Elasticum in the first week; and 5.80 ± 0.10 N for PET, 5.07 ± 0.81 N for ELATENS and 4.70 ± 0.26 N for Elasticum in the fourth week. However, there was no significant difference in the pullout strength required among the three thread types (Figure 2C).

### Thickness of the capsule

Capsules were formed around the suture threads inserted into the dorsal skin and subcutaneous tissue of rats. The thickness of the capsules was evaluated using histological analysis at weeks 1, 2 and 4.

**Figure 3.**
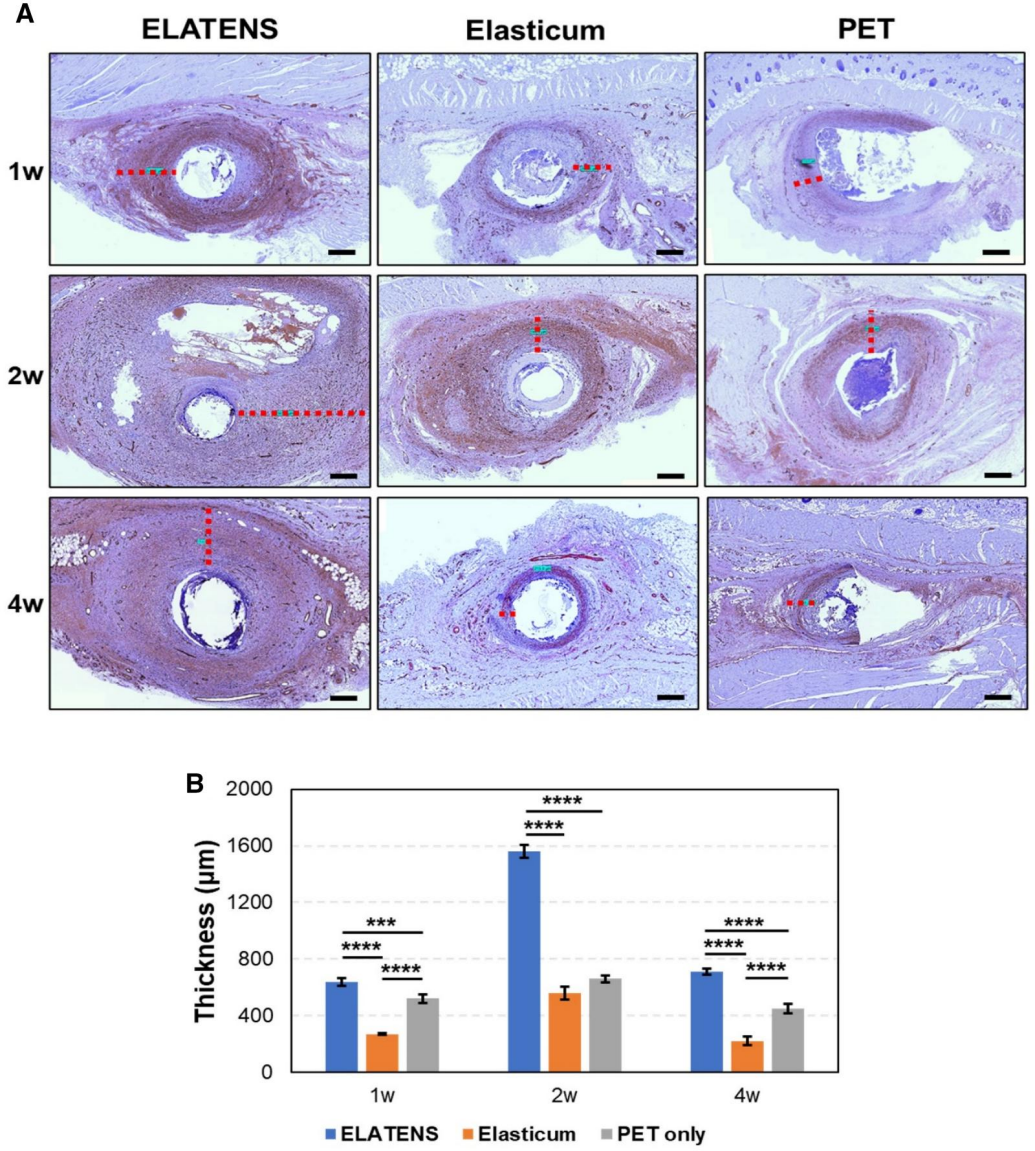
Comparison of the thickness of the capsule formed around the thread. (**A**, **B**) α smooth muscle actin staining of tissues implanted with ELATENS, Elasticum and PET threads. Tissue samples of the dorsal subcutaneous tissue were harvested and stained with α smooth muscle actin. Histologic analysis was performed at weeks 1, 2 and 4 after implantation with ELATENS, Elasticum and PET threads. Threads are depicted as white, empty circular regions. Representative images are shown. Scale bar = 500 μm.

The thickness of the capsules formed in week 1 was 640 ± 26.46 µm for ELATENS, 270 ± 5 µm for Elasticum and 520 ± 30 µm for PET. In week 2, the thickness was 1560 ± 45.83 µm for ELATENS, 560 ± 45.83 µm for Elasticum and 660 ± 26.46 µm for PET. And in week 4, the thickness was 710 ± 20 µm for ELATENS, 220 ± 30 µm for Elasticum and 450 ± 36.06 µm for PET (Figure 3). The comparison between groups was statistically significant (*P* < 0.001). Compared with other thread types, the thickness of the capsule formed around the implant area was the highest for ELATENS at all-time points. Further, capsule thickness was the highest at week 2 for all thread types, which decreased thereafter in the same pattern for all thread types. Capsule formation induced by the implanted suture thread may be involved in maintaining the length of the pulled skin. Hence, it can be substantiated that ELATENS is the most effective thread for pulling the skin.

### *Ex-vivo* studies on the frictional behavior of the thread

**Figure 4.**
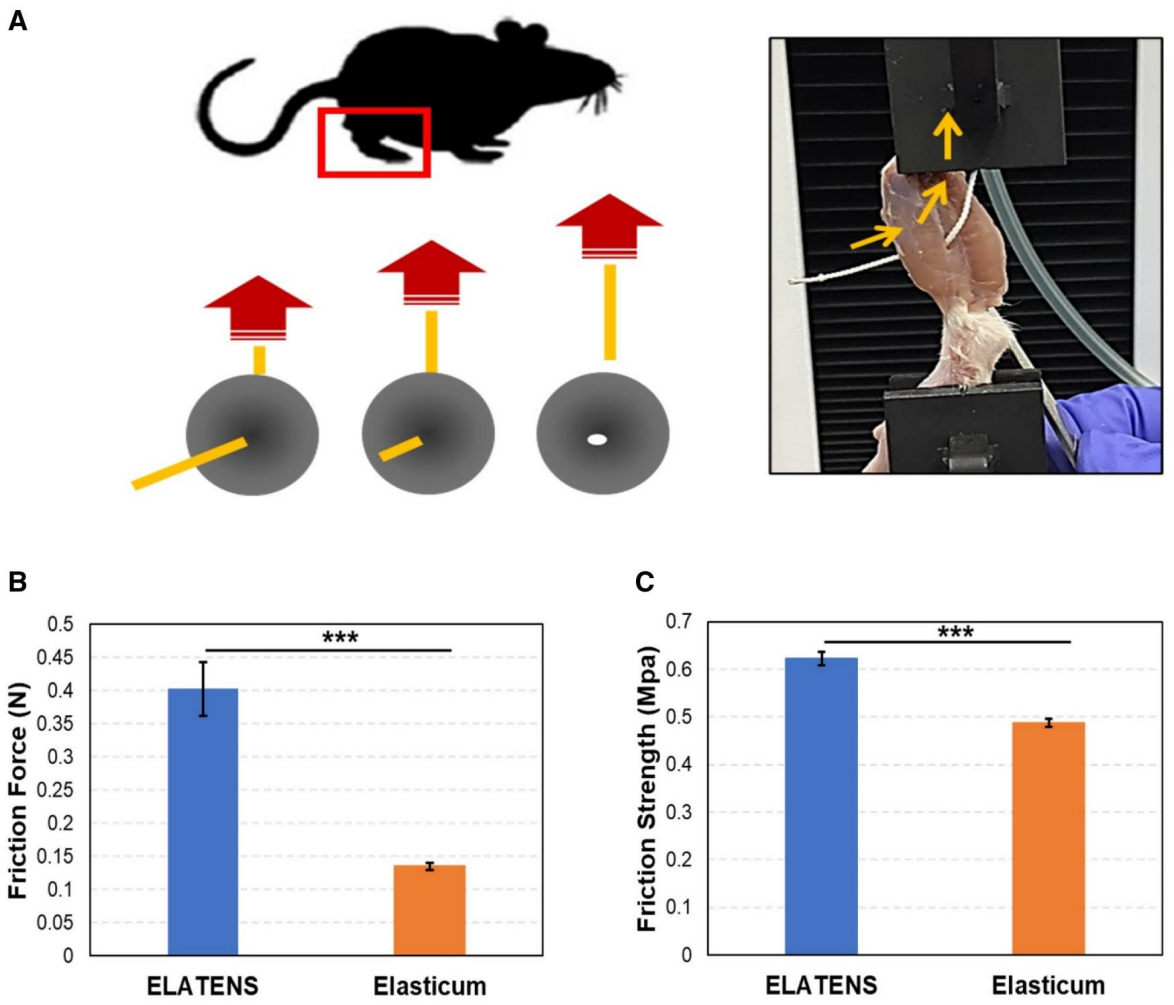
*Ex-vivo* studies on the frictional behavior of the thread inserted in the skeletal muscles of rats. Frictional properties of ELATENS and Elasticum were compared *ex-vivo*. (**A**) Schematic diagram of the experiment. (**B**) The average friction force values of ELATENS and Elasticum were compared. (**C**) The average friction strength values of ELATENS and Elasticum were compared.

The amount of friction produced by the thread against muscles was determined when one end of the suture thread inserted into the leg muscles of rats was pulled using a tensile tester (Figure 4A).

A comparison of the frictional force (N) and frictional strength (MPa) produced while pulling the thread revealed that ELATENS produced 0.40 ± 0.04 N and 0.62 ± 0.01 MPa, respectively, whereas Elasticum produced 0.14 ± 0.01 N and 0.49 ± 0.01 MPa, respectively (Figure 4B and C). The comparison between groups was statistically significant (*P* < 0.001). Hence, ELATENS produced more friction against the muscle tissues than Elasticum.

### Histopathological analysis—inflammatory response (CD3 and CD163)

Inflammatory cells were histologically evaluated at weeks 1, 4 and 8 after the suture thread was inserted into the dorsal skin and subcutaneous tissue of rats.

**Figure 5.**
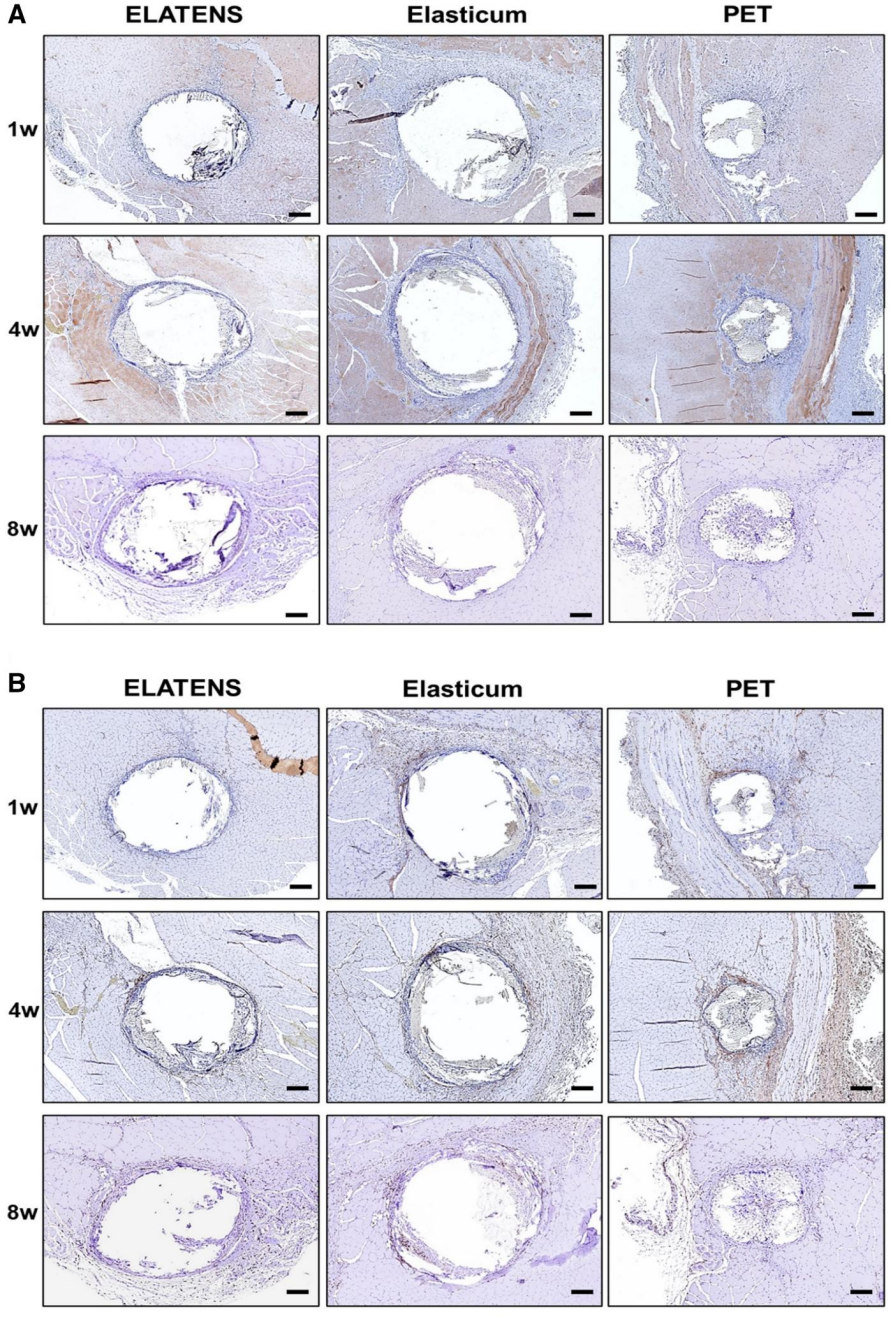
IHC analysis of inflammation using CD3 and CD163 markers around the tissues implanted with a thread. (**A**) Anti-CD3 and (**B**) anti-CD163 staining for inflammation response of tissues implanted with ELATENS, Elasticum and PET threads. Tissue samples of the dorsal subcutaneous tissue were harvested and stained with anti-CD3 and anti-CD163 antibodies. Histologic analysis was performed at weeks 1, 4 and 8 after implantation with ELATENS, Elasticum and PET threads. Threads are depicted as white, empty circular regions. Representative images are shown. Scale bar = 200 μm.

CD3 is a marker of inflammatory cells, and CD163 is a marker of type 2 macrophages. At weeks 1 and 4, inflammatory cell deposition increased similarly around the implant areas of ELATENS, Elasticum and PET, but at week 8, it showed a decreasing tendency (Figure 5).

### Histopathological analysis—the area of collagen formation (Masson’s trichrome and Herovici’s collagen staining)

Collagen formation was histologically evaluated at weeks 1, 4 and 8 after the suture thread was inserted into the dorsal skin and subcutaneous tissue of rats.

**Figure 6.**
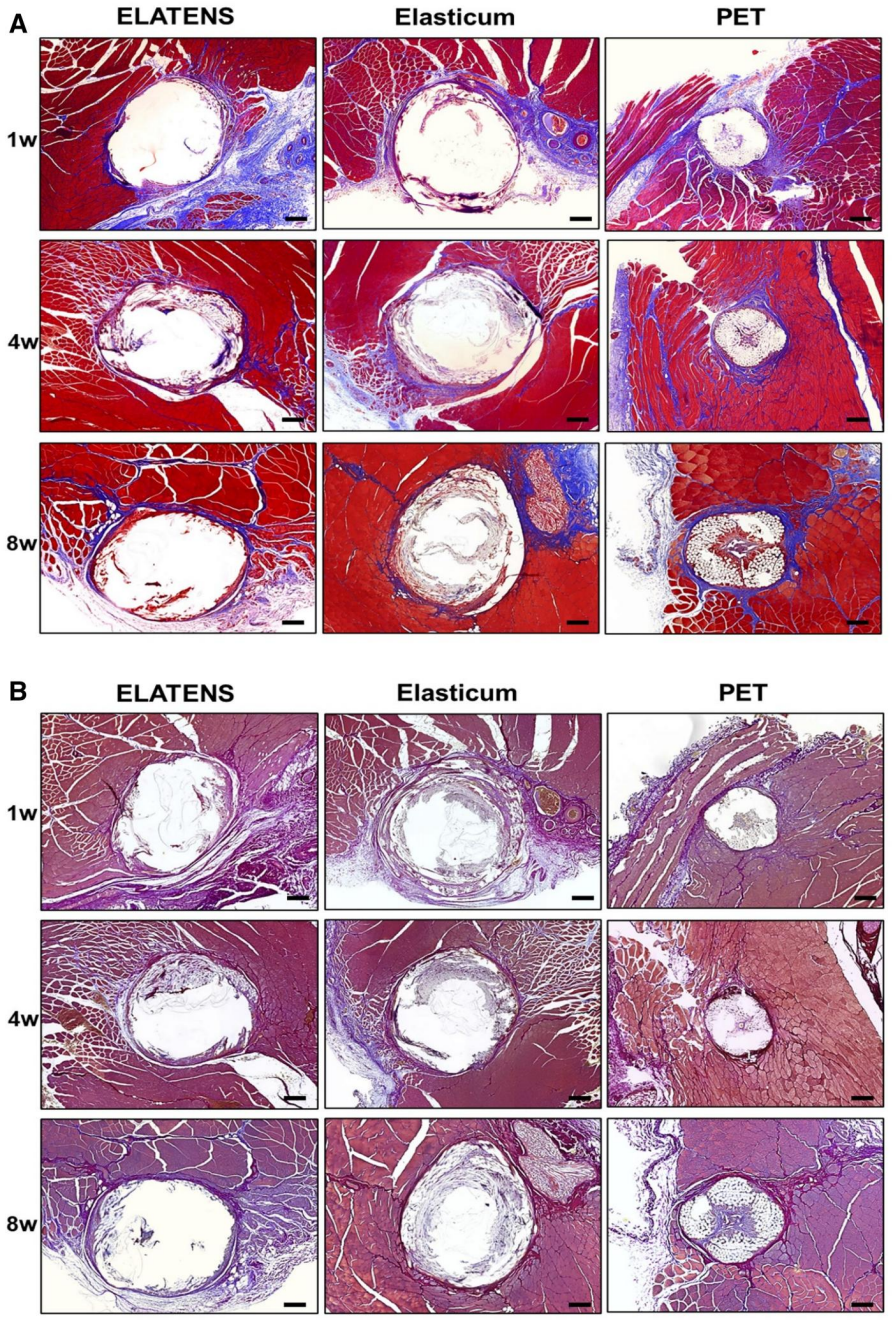
Staining of collagen via Masson trichrome and Herovici’s staining around the tissues implanted with a thread. (**A**) Masson’s trichrome and (**B**) Herovici’s staining for collagen formation of tissues implanted with ELATENS, Elasticum and PET threads. Tissue samples of the dorsal subcutaneous tissue were harvested and stained with Masson’s trichrome and Herovici’s. Collagen fibers were stained in blue by Masson’s trichrome staining. Type 1 collagen fibers were stained in purple and type 3 collagen fibers were stained in blue by Herovici’s staining. Histologic analysis was performed at weeks 1, 4 and 8 after implantation with ELATENS, Elasticum and PET threads. Threads are depicted as white, empty circular regions. Representative images are shown. Scale bar = 200 μm.

Collagen fibers were stained in blue by Masson’s trichrome staining. Type 1 collagen fibers were stained in purple and type 3 collagen fibers were stained in blue by Herovici’s staining. In terms of the amount of collagen formed around Elasticum and ELATENS, it was found that similar levels of collagen were formed for the two thread types at different time points (Figure 6).

## Discussion

In this study, to improve wrinkles caused by loss of skin elasticity due to aging, the effect of wrinkle improvement using sutures with elasticity of polymer materials was confirmed. Through this, we would like to suggest a new surgical procedure. The dorsal skin and subcutaneous tissue of the rat were pulled in a wrinkled state using a suture or other suture having elasticity of a polymer material, and the degree of skin retention was measured. The presence or absence of elasticity of a suture in a tissue with a lot of movement and severe wrinkle formation due to aging, such as facial skin, may be related to fixing the tissue and maintaining natural movement of the facial skin. In this study, histological evaluation and physical evaluation were conducted based on the comparison of the presence or absence of elasticity of sutures and the comparison of sutures with elasticity in terms of wrinkle improvement.

In terms of wrinkle improvement, according to a study by Jang *et al.*, when threads with and without cogs were implanted into the back skin of rats and histological evaluation was performed, more capsules were found in the threads with cogs. It has been reported that such encapsulation can induce stimulation for the formation of myofibroblasts and promote the formation of new skin tissue for wrinkle improvement [22]. In addition, the effective significance of the formation of the capsule is that it has been reported that the formation of capsule can support prolonged period skin and tissue tension [8], meaning that the thick capsule formation induced by the implant can maintain the length of the retracted skin.

In this study, elastic sutures and other sutures were inserted into the rat’s back skin and subcutaneous tissue at regular intervals. The wrinkled skin condition was induced by pulling the inserted thread, and the degree of stretching of the thread over time was measured to compare the degree of stretching maintenance. In *in-vivo* experiments using rats, ELATENS pulled the skin with a larger force than Elasticum, and a thick capsule was formed around the implant site at weeks 1, 2 and 4. In addition, in *ex-vivo* experiments with rats, it was found that, compared with Elasticum, a greater amount of friction was instantaneously produced against the muscle when ELATENS was pulled out. This suggests that immediately after suture insertion, the surface of ELATENS pulls the skin tissue, and with time, a thick capsule is formed around the surface of ELATENS, which can maintain the length of the pulled skin constantly. The difference in these results is due to the weaving method of the multifilament that forms the outer of the suture. Compared to Elasticum, which is a type weave in one direction, the outer pattern of ELATENS, a symmetrical braiding pattern, forms a dense structure. It is believed that the dense structure provides for encapsulation in a pattern favorable to cell migration or adhesion. In conclusion, the length of the skin pulled by ELATENS was well-maintained from immediately after suture insertion to week 4. Additionally, pullout strength after thread implantation and collagen formation around the implant site were similar, suggesting that they do not greatly affect the process of maintaining the length of skin tissue.

These results can also be found in papers that studied the surface morphology of threads used for lifting. Kapicioğlu *et al.*’s paper reported the conclusion that threads with cogs on the surface were effective in improving wrinkles by increasing dermal thickness and stimulating collagen production. ELATENS is a symmetrical braided pattern. It is believed to have a similar function to a cog by forming a dense structure in the axial direction and having a constant groove pattern [23]. In other words, the surface of the thread due to the symmetrical braided pattern acts as a kind of cog, causing greater friction at the insertion site, which is expected to induce further skin and tissue contraction.

We identified the biomechanical properties and biocompatibility of these special suture threads in the previous studies [16]. The tensile strength of the two suture threads of equal size was similar (50.83 ± 0.89 N in ELATENS and 49.97 ± 0.01 N in Elasticum^®^), whereas, in terms of Young’s modulus, ELATENS showed a higher value (0.81 ± 0.02 MPa in ELATENS and 0.57 ± 0.01 MPa in Elasticum^®^) than the other threads. These results of ELATENS’s biomechanical properties can also support the result of *in vivo* skin lifting. It can be assumed that higher Young’s modulus of ELATENS can lift skin tissues effectively and that the skin tissues will be constantly maintained at high tensile strength.

These characteristics can be seen as advantages that elastic thread can have in lifting, similar to the study published by OH *et al.* OH *et al.* report that elastic thread is suitable for buttock lifting due to its strength in pulling sagging skin tissue and natural movement. By confirming that ELATENS can maintain the tensile strength that pulls tissue and the deformation of force due to tissue movement, it can be judged to have the functionality to maintain natural lifting [15].

It is known that the force on the dorsal skin layer of rats is weaker than that on the skin layer of humans [18]. Further, rat dorsal skin is prone to movement, and the experiments were carried out with the insertion of only one thread. Thus, the results described herein may differ from the effects of thread lifting on humans. Therefore, further studies are warranted to validate this method’s applicability in clinical settings.

Through this study, it was determined that non-absorbable elastic thread can effectively pull and fix skin tissue, and can easily lift sagging skin tissue by continuously maintaining high tensile strength. In addition, the degree of encapsulation between tissues and the degree of new collagen fiber formation for improving skin wrinkles were excellent. It is believed that the more the surface structure of the thread has a certain pattern of roughness, the more it promotes intercellular stimulation or inflow into wrinkled tissue, thereby promoting collagen formation.

## Supplementary Material

rbae069_Supplementary_Data

## References

[1] Karimi K , Reivitis A. Lifting the lower face with an absorbable polydioxanone (PDO) thread. J Drugs Dermatol 2017;16:932–4. 28915290

[2] Baker D. Rhytidectomy with lateral SMASectomy. Facial Plast Surg 2000;16:209–13. 11802569 10.1055/s-2000-13591

[3] Liu TS , Owsley JQ. Long-term results of face lift surgery: patient photographs compared with patient satisfaction ratings. Plast Reconstr Surg 2012;129:253–62. 22186515 10.1097/PRS.0b013e3182362b55

[4] Hwang K , Kim H, Kim DJ. Thickness of skin and subcutaneous tissue of the free flap donor sites: a histologic study. Microsurgery 2016;36:54–8. 26529557 10.1002/micr.30000

[5] Byun JS , Hwang K, Lee SY, Song JM, Kim H. Forces required to pull the superficial fascia in facelifts. Plast Surg (Oakv) 2018;26:40–5. 29619358 10.1177/2292550317747853 PMC5871114

[6] Sulamanidze MA , Fournier PF, Paikidze TG, Sulamanidze GM. Removal of facial soft tissue ptosis with special threads. Dermatol Surg 2002;28:367–71. 12030865 10.1046/j.1524-4725.2002.01297.x

[7] Khiabanloo RS , Jebreili R, Aalipour E, Eftekhari H, Saljoughi N, Shahidi A. Innovative techniques for thread lifting of face and neck. J Cosmet Dermatol 2019;18:1846–55. 31050152 10.1111/jocd.12969

[8] Kurita M , Matsumoto D, Kato H, Araki J, Higashino T, Fujino T, Takasu K, Yoshimura K. Tissue reactions to cog structure and pure gold in lifting threads: a histological study in rats. Aesthet Surg J 2011;31:347–51. 21385746 10.1177/1090820X11398474

[9] Kim BJ , Choi JH, Lee Y. Development of facial rejuvenation procedures: thirty years of clinical experience with face lifts. Arch Plast Surg 2015;42:521–31. 26430622 10.5999/aps.2015.42.5.521 PMC4579162

[10] Vidal MA , Mendes Junior CEDS, Sanches JA. Elastic suture: an alternative for extensive skin loss. Brazil J Plast Surg 2014;29:146.

[11] Foumenteze JP , Guillo D, Jeanblanc G. *Multi-Centric Retrospective Clinical Study for Suspension Thread Spring Thread™*. Somerefs, 2010. https://easyfairsassets.com/sites/188/2021/05/Someref-retrospective-study.2008-2010-1.pdf (23 August 2011, date last accessed).

[12] Celik N. The new era in office-based facial rejuvenation: promising technology of silicone threads. Front Life Sci RT 2021;2:30–4.

[13] Kang MS , Shin JS, Nam SM, Park ES. Evaluation of elastic lift for facial rejuvenation. Arch Aesthetic Plast Surg 2016;22:20–7.

[14] Kang MS , Kim SH, Nam SM, Park ES. Evaluation of elastic lift for neck rejuvenation. Arch Aesth Plast Surg 2016;22:68–73.

[15] Oh CH , Jang SB, Kang CM, Shim JS. Buttock lifting using elastic thread (Elasticum^®^) with a new classification of gluteal ptosis. Aesth Plast Surg 2018;42:1050–8. 10.1007/s00266-018-1124-z 29610954

[16] Choi Y , Kang M, Choi MS, Song JK, Lih E, Lee D, Jung HH. Biomechanical properties and biocompatibility of a non-absorbable elastic thread. J Funct Biomater 2019;10:51. 31744160 10.3390/jfb10040051 PMC6963933

[17] Selvi F , Cakarer S, Can T, Kirli TOPCU SI, Palancioglu A, Keskin B, Bilgic B, Yaltirik M, Keskin C. Effects of different suture materials on tissue healing. J Istanb Univ Fac Dent 2016;50:35–42. 28955553 10.17096/jiufd.79438 PMC5573451

[18] Marchant LH , Knapp S, Apter JT. Effect of elongation rate on tensile strength of surgical suture materials. Surg Gynecol Obstet 1974;139:231–3. 4601701

[19] Zaruby J , Gingras K, Taylor J, Maul D. An in vivo comparison of barbed suture devices and conventional monofilament sutures for cosmetic skin closure: biomechanical wound strength and histology. Aesthet Surg J 2011;31:232–40. 21317121 10.1177/1090820X10395010

[20] ASTM D2256. Standard test method for tensile properties of yarns by the single-strand method, 2010.

[21] Abellán D , Nart J, Pascual A, Cohen RE, Sanz-Moliner JD. Physical and mechanical evaluation of five suture materials on three knot configurations: an in vitro study. Polymers (Basel) 2016;8:147. 30979247 10.3390/polym8040147 PMC6432448

[22] Jang HJ , Lee WS, Hwang K, Park JH, Kim DJ. Effect of cog threads under rat skin. Dermatol Surg 2005;31:1639–43; discussion 1644. 16336880 10.2310/6350.2005.31301

[23] Kapicioğlu Y , Gül M, Saraç G, Yiğitcan B, Gözükara H. Comparison of antiaging effects on rat skin of cog thread and poly-l-lactic acid thread. Dermatol Surg 2019;45:438–45. 30608294 10.1097/DSS.0000000000001717

