# Supplementary material for: Comparison of tissue response and lifting effect induced by non-absorbable elastic thread and commercialized threads in rat model
Source: Regen Biomater. 2024 Jun 17;11:rbae069. doi: 10.1093/rb/rbae069 (PMC11272176; doi:10.1093/rb/rbae069)
Supplement: rbae069_Supplementary_Data [file rbae069_supplementary_data.docx]

**Supplementary figure**

**
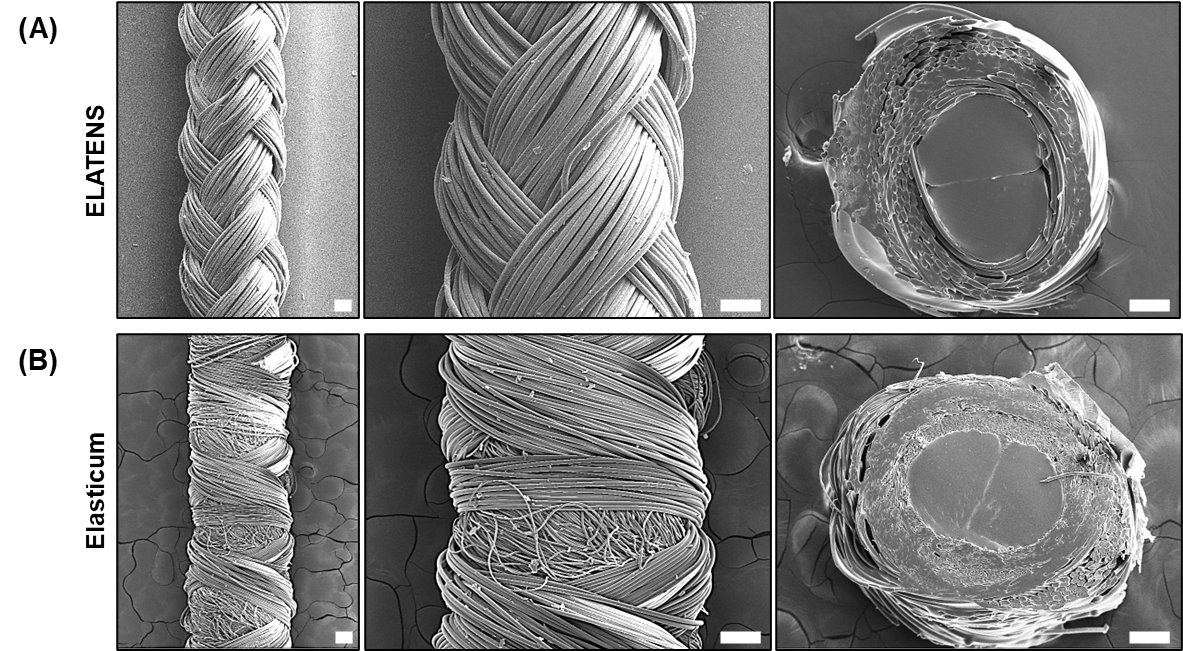
**

**Supplementary figure 1. SEM image of shapes and cross-section of threads.**

(A) ELATENS. (B) Elasticum. Representative images are shown. Scale bar = 200 ㎛.
